# ﻿Redelimitation of *Heteroradulum* (Auriculariales, Basidiomycota) with *H.australiense* sp. nov.

**DOI:** 10.3897/mycokeys.86.76425

**Published:** 2022-01-19

**Authors:** Qian-Zhu Li, Shi-Liang Liu, Xue-Wei Wang, Tom W. May, Li-Wei Zhou

**Affiliations:** 1 State Key Laboratory of Mycology, Institute of Microbiology, Chinese Academy of Sciences, Beijing, 100101, China Institute of Microbiology, Chinese Academy of Sciences Beijing China; 2 Institute of Applied Ecology, Chinese Academy of Sciences, Shenyang, 110016, China University of Chinese Academy of Sciences Beijing China; 3 University of Chinese Academy of Sciences, Beijing, 100049, China Institute of Applied Ecology, Chinese Academy of Sciences Shenyang China; 4 Royal Botanic Gardens Victoria, Birdwood Avenue, Melbourne, 3004, Australia Royal Botanic Gardens Victoria Melbourne Australia

**Keywords:** *Agaricomycetes*, Australia, *Grammatus*, heterobasidiomycetes, two new taxa, wood-inhabiting fungi

## Abstract

*Auriculariales* accommodates species with diverse basidiomes and hymenophores. From morphological and phylogenetic perspectives, we perform a taxonomic study on *Heteroradulum*, a recently validated genus within the *Auriculariales*. The genus *Grammatus* is merged into *Heteroradulum*, and thus its generic type *G.labyrinthinus* is combined with *Heteroradulum* and *G.semis* is reaccepted as a member of *Heteroradulum*. *Heteroradulumaustraliense* is newly described on the basis of three Australian specimens. *Heteroradulumyunnanense* is excluded from this genus and its taxonomic position at the generic level is considered uncertain. Accordingly, the circumscription of *Heteroradulum* is re-delimited and the concept of this genus is adjusted by including irpicoid to poroid hymenophores and a hyphal system with clamp connections or simple septa. A key to all nine accepted species of *Heteroradulum* is presented.

## ﻿Introduction

*Auriculariales* (*Agaricomycetes*, *Basidiomycota*) is characterized by a wood-inhabiting habit and longitudinally or transversely septate basidia (Weiß and Oberwinkler 2001). While the type genus *Auricularia* Bull. and a number of additional genera accommodate “jelly fungi” with gelatinous basidiomes, some other genera in this order have tough basidiomes with smooth, hydnoid, poroid or lamellate hymenophores (Weiß and Oberwinkler 2001; Zhou and Dai 2013; Malysheva and Spirin 2017; Malysheva et al. 2018). The diverse macromorphological characters result in the taxonomy of *Auriculariales* having rarely focused on the whole order. Therefore, within this order, the intergeneric relationships, viz. their taxonomic positions at the family level, are not clear; moreover, the independence and monophyly of certain genera still needs to be addressed (Zhou and Dai 2013; Malysheva and Spirin 2017).

Weiß and Oberwinkler (2001) performed the first comprehensive phylogenetic analysis of *Auriculariales*. The redefined *Auriculariales* was composed of five well supported groups, but the monophyly of this order even as represented by limited samples was not statistically supported (Weiß and Oberwinkler 2001). With this phylogenetic frame as a main reference, the taxonomy and phylogeny of poroid and lamellate species were further explored (Miettinen et al. 2012; Zhou and Dai 2013; Sotome et al. 2014; Wu et al. 2017; Spirin et al. 2019a). In addition, the knowledge of the diversity of species with gelatinous basidiomes has been extremely enriched recently (Bandara et al. 2015; Wu et al. 2015a, b; Malysheva et al. 2018; Spirin et al. 2018, 2019b; Chen et al. 2020; Ye et al. 2020; Wang and Thorn 2021).

On the basis of morphology, the non-gelatinous species of *Auriculariales* that are resupinate with or without a narrow reflexed pileus (i.e., corticioid or stereoid) have been placed in the genera *Eichleriella* Bres., *Exidiopsis* (Bref.) Möller and *Heterochaete* Pat. (Bodman 1952; Wells 1961; Wells and Raitviir 1977, 1980). Circumscriptions of the genera changed over time, but according to Wells and Raitviir (1977, 1980) the distinguishing character of *Eichleriella* was the presence of a basal layer of thick-walled, brown hyphae, while the delimitation of *Heterochaete* relied on the presence of minute, sterile spines (hyphal pegs) on the hymenophore. With the integration of molecular data into phylogenies including these and related genera, *Hirneolina* (Pat.) Bres. and *Tremellochaete* Raitv. have been reinstated and a number of novel genera have been introduced, including *Adustochaete* Alvarenga & K.H. Larss., *Amphistereum* Spirin & Malysheva, *Crystallodon* Alvarenga (Alvarenga and Gibertoni 2021), *Heteroradulum* Lloyd ex Spirin & Malysheva, *Proterochaete* Spirin & Malysheva and *Sclerotrema* Spirin & Malysheva (Malysheva and Spirin 2017; Alvarenga et al. 2019). After transfer of some species to these novel genera, *Eichleriella* (as far as sequenced species go) is monophyletic, but *Exidiopsis* is currently polyphyletic. The only species remaining in *Heterochaete* for which sequences are available is the type (*H.andina* Pat. & Lagerh.) and this is close to the type of *Exidiopsis* [*E.effusa* (Bref. ex Sacc.) Möller], leading Malysheva and Spirin (2017) to suggest that the two genera may be synonymous. Numerous species remain in *Heterochaete* that are yet to be sequenced, while those that have been sequenced, apart from *H.andina*, are placed in *Crystallodon*, *Eichleriella* and *Heteroradulum*.

*Heteroradulum*, typified by *H.kmetii* (Bres.) Spirin & Malysheva, was validated by Malysheva and Spirin (2017), who included seven species in this genus. Later, the new genus *Grammatus* H.S. Yuan & Decock was introduced, typified by *G.labyrinthinus* H.S. Yuan & Decock, and *H.semis* was transferred to *Grammatus* (Yuan et al. 2018). However, the phylogenetic analysis of Yuan et al. (2018) did not recover a monophyletic group for the remaining sampled species of *Heteroradulum*. Recently, *Heteroradulumyunnanense* C.L. Zhao (as ‘*yunnanensis*’) was newly described in *Heteroradulum* (Guan et al. 2020) but the phylogeny sampled only *Heteroradulum* as ingroup taxa and the analysis cannot properly determine whether *H.yunnanense*, which had a basal phylogenetic position, belongs to *Heteroradulum* or not. Therefore, questions remain about the delimitation of *Heteroradulum* from a phylogenetic perspective.

During field trips in Australia, three specimens bearing corticioid basidiomes and longitudinally septate basidia were collected. Based on these specimens, a new species of *Heteroradulum* was identified and is presented below along with a revised phylogeny of the genus and its relatives based on molecular data. This phylogenetic analysis leads to a revised circumscription of *Heteroradulum*.

## ﻿Materials and methods

### ﻿Morphological examination

The studied specimens are preserved at the Fungarium, Institute of Microbiology, Chinese Academy of Sciences (HMAS), Beijing, China and the National Herbarium of Victoria (MEL), Melbourne, Australia. The hymenial surfaces of basidiomes were observed and photographed with the aid of a stereomicroscope (LEICA M125). Special color terms follow Petersen (1996). Microscopic procedure followed Wang et al. (2020). A Nikon Eclipse 80i light microscope (Tokyo, Japan) was used at magnifications up to 1000×. Specimen sections were prepared with Cotton Blue (CB), Melzer’s reagent (IKI) and 5% potassium hydroxide (KOH) for observation. All measurements were taken from materials mounted in CB. Drawings were made with the aid of a drawing tube. When presenting the variation of basidiospore sizes, 5% of the measurements were excluded from each end of the range and are given in parentheses. The following abbreviations are used in the text: L = mean basidiospore length (arithmetic average of all measured basidiospores), W = mean basidiospore width (arithmetic average of all measured basidiospores), Q = variation in the L/W ratios between the specimens studied, and (a/b) = number of basidiospores (a) measured from given number (b) of specimens.

### ﻿Molecular sequencing

Crude DNA was extracted from basidiomes of dry specimens using FH Plant DNA Kit (Beijing Demeter Biotech Co., Ltd., Beijing, China), and then directly used as template for subsequent PCR amplifications. The primer pairs ITS5/ITS4 (White et al. 1990) and LR0R/LR7 (Vilgalys and Hester 1990) were selected for amplifying the ITS and nLSU regions, respectively. The PCR procedures are as follows: for the ITS region, initial denaturation at 95 °C for 3 min, followed by 34 cycles at 94 °C for 40 s, 57.2 °C for 45 s and 72 °C for 1 min, and a final extension at 72 °C for 10 min, while for the nLSU region, initial denaturation at 94 °C for 1min, followed by 34 cycles at 94 °C for 30 s, 47.2 °C for 1 min and 72 °C for 1.5 min, and a final extension at 72 °C for 10 min. The PCR products were sequenced with the same primers as those used in amplifications at the Beijing Genomics Institute, Beijing, China. The newly generated sequences were deposited in GenBank (https://www.ncbi.nlm.nih.gov/genbank/; Table 1).

**Table 1.** Information on species and specimens used in the phylogenetic analysis. The newly generated sequences are in boldface. Type specimens are indicated with an asterisk (*).

Species	Voucher number	GenBank accession number	
		ITS	nLSU
*Amphistereumleveilleanum*	FP-106715	KX262119	KX262168
*A.schrenkii*	HHB8476	KX262130	KX262178
*Aporpiumhexagonoides*	ML297	AB871754	AB871735
*Auriculariamesenterica*	FO25132	AF291271	AF291292
*A.mesenterica*	TUFC12805	AB915192	AB915191
*A.polytricha*	TUFC12920	AB871752	AB871733
*Basidiodendroneyrei*	TUFC14484	AB871753	AB871734
*Eichleriellabactriana*	TAAM55071*	KX262121	KX262170
*E.leucophaea*	LE303261	KX262111	KX262161
*Elmerinacaryae*	WD2207	AB871751	AB871730
*E.foliacea*	Yuan 5691	JQ764666	JQ764644
*E.hispida*	WD548	AB871768	AB871749
	E701	AB871767	AB871748
*Exidiaglandulosa*	TUFC34008	AB871761	AB871742
*E.glandulosa*	MW355	AF291273	AF291319
*E.pithya*	MW313	AF291275	AF291321
*Exidiopsiscalcea*	MW331	AF291280	AF291326
*E.effusa*	OM19136	KX262145	KX262193
*E.grisea E.grisea*	RoKi162	AF291281	AF291328
	TUFC100049	AB871765	AB871746
*Exidia* sp.	TUFC34333	AB871764	AB871745
	FO46291	AF291282	AF291329
*Heteroradulumadnatum*	LR23453*	KX262116	KX262165
***H.australiense***	LWZ 20180512–20*	**MZ325254**	**MZ310424**
	LWZ 20180512–25*	**MZ325255**	**MZ310425**
	LWZ 20180515–26*	**MZ325256**	**MZ310426**
*H.deglubens*	FO12006	AF291272	AF291318
	LE38182	KX262112	KX262162
	LE225523	KX262113	KX262163
	TAAM064782	KX262101	KX262148
	Solheim1864	KX262133	KX262181
*H.kmetii*	Kmet*	KX262124	KX262173
	VS8858	KX262105	KX262154
	VS8864	KX262106	KX262155
	VS8981	KX262132	KX262180
	VS8988	KX262107	KX262156
	LE38181	KX262109	KX262159
	DAOM145605	KX262135	KX262183
	DAOM31292	KX262134	KX262182
	OF-295640	KX262122	KX262171
	OF-295641	KX262117	KX262166
*H.kmetii*	OF-295639	KX262128	KX262177
	VS7967	KX262108	KX262157
	TAAM9847	KX262125	KX262174
	VS6466	KX262104	KX262152
	LE303456	KX262103	KX262151
	TAAM149179	KX262102	KX262149
	CWU4563	KX262127	KX262176
	CWU6152	KX262126	KX262175
	LR14389	KX262131	KX262179
*H.labyrinthinum*	Yuan 1759*	KM379137	KM379138
	Yuan 1600*	KM379139	KM379140
*H.semis*	OM10618*	KX262146	KX262194
*H.yunnanense*	CLZhao 4023*	MT215568	MT215564
	CLZhao 8106*	MT215569	MT215565
	CLZhao 9132*	MT215570	MT215566
	CLZhao 9200*	MT215571	MT215567
*Heteroradulum* sp.	USJ55639	AF291285	AF291336
*Hirneolinahirneoloides*	USJ55480	AF291283	AF291334
*Sclerotremagriseobrunneum*	VS7674	KX262140	KX262188
*Sistotremabrinkmannii*	236	JX535169	JX535170
*Tremellochaetejaponica*	LE303446	KX262110	KX262160

### ﻿Phylogenetic analysis

Besides the newly sequenced specimens, additional taxa representing all main lineages within the *Auriculariales* were also included in the current phylogenetic analysis, and *Sistotremabrinkmannii* (Bres.) J. Erikss. within the *Cantharellales* was selected as an outgroup taxon following Malysheva and Spirin (2017) (Table 1). The datasets of ITS and nLSU regions were aligned separately using MAFFT version 7 (Katoh and Standley 2013) with the G-INS-i strategy (Katoh et al. 2005). Then, the two resulting alignments were concatenated as a single alignment for subsequent phylogenetic analysis. This alignment was submitted to TreeBASE (http://www.treebase.org; accession number S28342) and its best-fit evolutionary model was estimated using jModelTest (Guindon and Gascuel 2003; Posada 2008) with calculation under the Akaike information criterion. Following the resulting evolutionary model SYM + I + G, Maximum Likelihood (ML) and Bayesian Inference (BI) analyses were performed. The ML analysis was conducted using raxmlGUI 1.2 (Silvestro and Michalak 2012; Stamatakis 2006) with the calculation of bootstrap (BS) replicates under the auto FC option (Pattengale et al. 2010). The BI analysis was conducted using MrBayes 3.2 (Ronquist et al. 2012) with two independent runs, each including four chains of 10 million generations and starting from random trees. Trees were sampled every 1000^th^ generation. The first 25% of the resulting trees was discarded as burn-in, while the remaining 75% were used for constructing a 50% majority consensus tree and calculating Bayesian posterior probabilities (BPPs). Chain convergence was determined using Tracer 1.5 (http://tree.bio.ed.ac.uk/software/tracer/).

## ﻿Results

**Figure 1.**
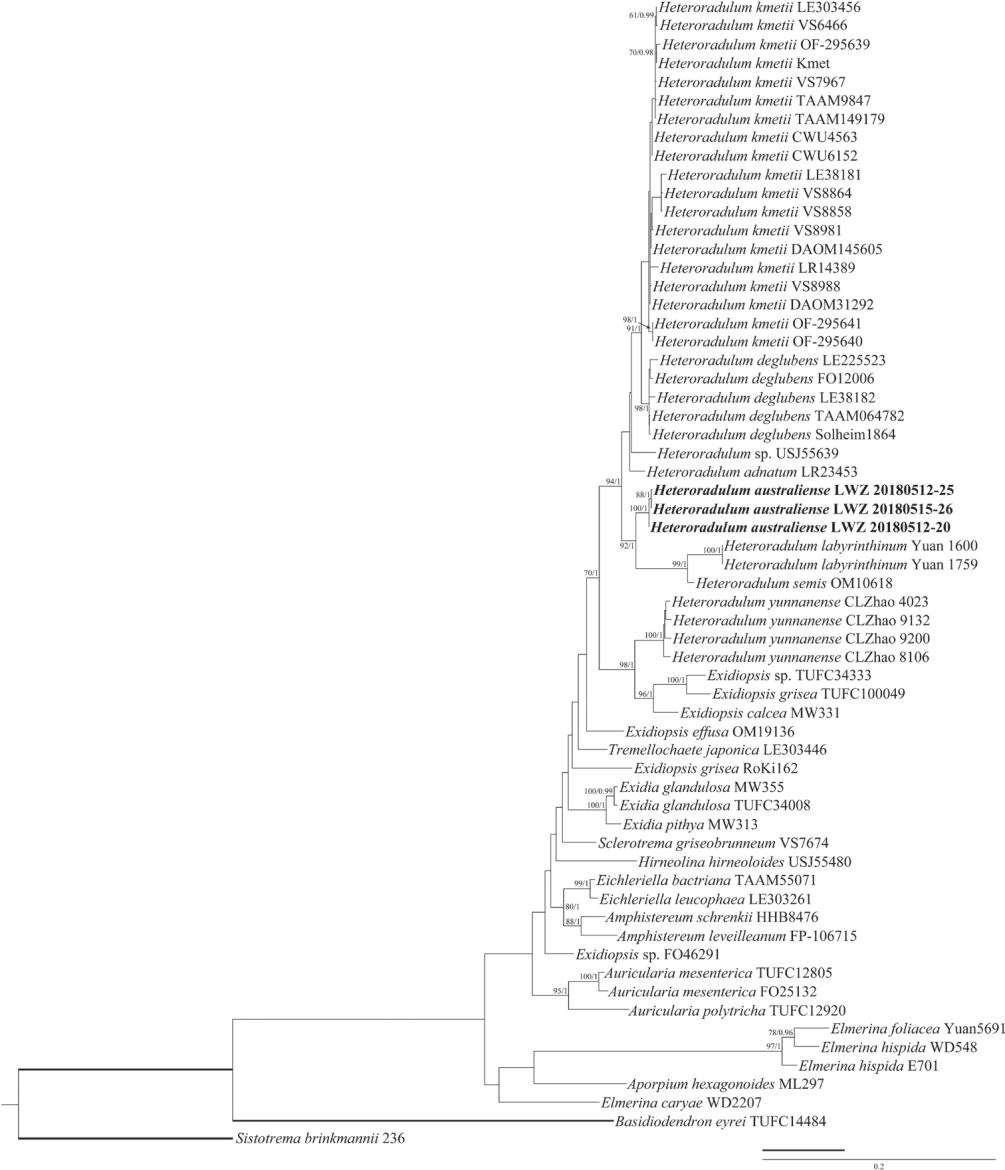
Phylogenetic delimitation of *Heteroradulum* within the *Auriculariales* inferred from the combined dataset of ITS and nLSU regions. The topology generated by the maximum likelihood analysis is presented along with bootstrap values and Bayesian posterior probabilities above 50% and 0.8, respectively, at the nodes. The specimens of the newly described species are in boldface.

Three ITS and three nLSU sequences were newly generated from three Australian specimens of *Heteroradulum* for this study. The alignment used for phylogenetic analysis has 62 collections and 1583 characters. The ML analysis ended after 300 BS replicates. The BI analysis converged after 10 million generations as indicated by an average standard deviation of split frequencies = 0.004375, the effective sample sizes of all parameters above 4960 and the potential scale reduction factors equal to 1.000. The ML and BI analyses generated similar topologies in main lineages, and thus the topology generated from ML analysis is presented along with BS values above 50% and BPPs above 0.8 at the nodes (Figure 1).

The current phylogeny groups *Grammatus* and *Heteroradulum*, with the exception of *H.yunnanense*, as a strongly supported clade (BS = 94%, BPP = 1; Figure 1). Within this clade, the three newly sequenced Australian specimens grouped as a fully supported lineage, as sister to the two species formerly placed in the genus *Grammatus*, forming a strongly supported subclade (BS = 92%, BPP = 1), while the monophyly of the subclade including the remaining species of *Heteroradulum*, viz. *H.adnatum* Spirin & Malysheva, *H.deglubens* (Berk. & Broome) Spirin & Malysheva and *H.kmetii*, did not receive reliable statistical support (Figure 1). This topology means that the subclades containing the types of *Grammatus* and *Heteroradulum* respectively are not reciprocally monophyletic within the strongly supported clade. *Heteroradulumyunnanense* falls outside of the *Heteroradulum* clade as a well-supported sister to a clade comprised of three taxa currently placed in *Exidiopsis* (Figure 1).

## ﻿Taxonomy

### ﻿ Heteroradulum

Taxon classificationFungiAuricularialesExidiaceae

Lloyd ex Spirin & Malysheva, in Malysheva & Spirin, Fungal Biology 121(8): 709 (2017)

145005D6-66A0-5FCA-A753-9036CF9ED846

= Grammatus H.S. Yuan & Decock, in Yuan, Lu & Decock, MycoKeys 35: 32 (2018)

#### Remarks.

Following the phylogenetic analysis, we treat *Grammatus* and *Heteroradulum* as a single genus, for which *Heteroradulum* has priority. The newly revealed Australian lineage is described as the new species *Heteroradulumaustraliense* below. In addition, *G.labyrinthinus* is combined to *Heteroradulum* and *G.semis* (Spirin & Malysheva) H.S. Yuan & Decock is reaccepted as a member of *Heteroradulum*.

Malysheva and Spirin (2017) defined the morphological characters of *Heteroradulum* according to the seven accepted species at that time, viz. *H.adnatum*, *H.brasiliense* (Bodman) Spirin & Malysheva, *H.deglubens*, *H.kmetii*, *H.lividofuscum* (Pat.) Spirin & Malysheva, *H.semis* and *H.spinulosum* (Berk. & M.A. Curtis) Spirin & Malysheva. The concept of this genus was adjusted by below including *H.australiense* with generative hyphae bearing a mixture of simple septa and clamp connections and *H.labyrinthinus* with irpicoid to poroid hymenophores.

### ﻿ Heteroradulum australiense

Taxon classificationFungiAuricularialesExidiaceae

L.W. Zhou, Q.Z. Li & S.L. Liu sp. nov.

7D2122F2-2C4A-56C3-BDA8-8F8D56DAC80B

842485

Figures 2 , 3

#### Etymology.

*australiense* (Lat.), refers to Australia.

#### Type.

Australia, Tasmania, Tahune Adventures, Arve River Picnic Area, on fallen angiosperm branch, 15 May 2018, L.W. Zhou, LWZ 20180515–26 (holotype in MEL, isotype in HMAS).

**Figure 2.**
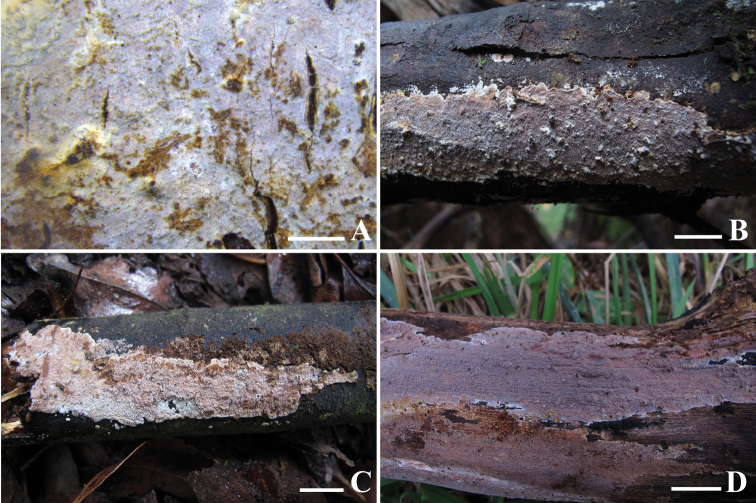
Basidiomes of *Heteroradulumaustraliense*. **A–B** LWZ 20180515–26 (holotype) **C** LWZ 20180512–20 (paratype) **D** LWZ 20180512–25 (paratype). Scale bars: 2 mm (**A**); 1 cm (**B–D**).

#### Diagnosis.

*Heteroradulumaustraliense* differs from other species in this genus by the generative hyphae having a mixture of simple septa and clamp connections.

**Figure 3.**
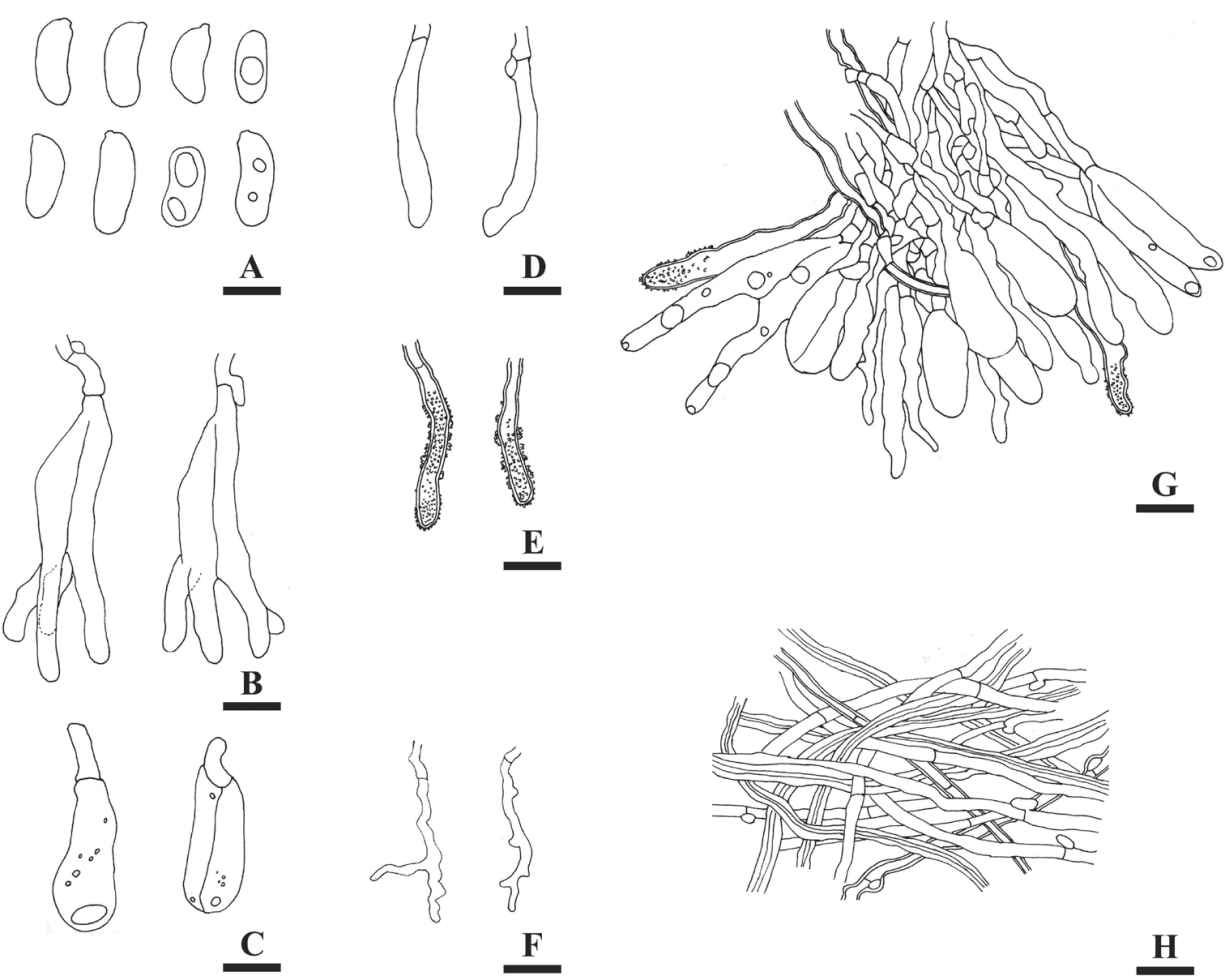
Microscopic structures of *Heteroradulumaustraliense* (drawn from the holotype, LWZ 20180515–26). **A** basidiospores **B, C** basidia and basidioles **D** cystidia **E** skeletocystidia **F** dendrohyphidia **G** hymenium **H** subicular hyphae. Scale bars: 10 μm (**A–H**).

#### Description.

Basidiomes annual, resupinate, adnate, without odor or taste when fresh, leathery, covering 24.5 cm in widest dimension and up to 0.4 mm thick. Hymenophore odontioid, covered by irregularly arranged spines, up to 0.2 mm long, 3–5 per mm, pale red to reddish lilac when fresh, pale orange to brownish gray upon drying. Margin smooth, adnate, yellowish white, 0.5 mm wide.

Hyphal system dimitic; generative hyphae with simple septa or clamp connections; skeletal hyphae IKI–, CB+; tissue unchanged in KOH. Subicular generative hyphae hyaline, thin to thick-walled, rarely branched, 2–4 μm in diam; skeletal hyphae hyaline to brownish, thick-walled, interwoven, occasionally branched, 2.5–4 μm in diam, sometimes irregularly inflated up to 6 μm. Subhymenial generative hyphae hyaline to brownish, thin-to slightly thick-walled, 2–3.5 μm in diam; skeletal hyphae brownish, thick-walled, encrusted by grainy crystals, subparallel and vertical along substrate, compact, 2–4.5 μm in diam. Clavate to subcylindrical cystidia abundant, septate with or without clamp connections, thin-walled, 24–56 × 3–8 μm. Skeletocystidia present as endings of subicular skeletal hyphae, distinctly thick-walled, heavily encrusted by grainy crystals, 4–7 μm in diam. Dendrohyphidia abundant, scattered among hymenial cells, covering the hymenial surface, branched, up to 54 μm long, 2–3 μm in diam. Basidia narrowly ovoid to obconical, longitudinally septate, four-celled, 29–34.5 × 10–13.5 μm, with enucleate stalk up to 14 × 4 μm. Basidiospores cylindrical, slightly or distinctly curved, hyaline, thin-walled, smooth, occasionally with oily inclusions, IKI–, CB–, (14.5–)15–20(–20.5) × 5–7(–7.5) μm, L = 17.0 μm, W = 6.2 μm, Q = 2.66–2.88 (n = 90/3).

#### Specimens (paratypes) examined.

Australia, Victoria, Yarra Ranges National Park, Dandenong Ranges Botanic Garden, on a fallen branch of *Eucalyptus*, 12 May 2018, L.W. Zhou, LWZ 20180512–20 (HMAS), on fallen angiosperm branch, 12 May 2018, L.W. Zhou, LWZ 20180512–25 (HMAS).

#### Remarks.

*Heteroradulumaustraliense* is characterized by pale red to reddish lilac basidiomes, a dimitic hyphal system, generative hyphae with simple septa or clamp connections, abundant skeletocystidia in the hymenium, and basidia with an enucleate stalk. *Heteroradulumkmetii* and *H.spinulosum* resemble *H.australiense* by odontoid hymenophores, a dimitic hyphal system and the presence of skeletocystidia (Malysheva and Spirin 2017). However, *H.kmetii* has longer spines (up to 1 mm long) and slightly larger basidiospores (14.3–22.3 × 6–9.2 μm), and generative hyphae always with clamp connections; and *H.spinulosum* differs by basidia with a shorter enucleate stalk (up to 6 μm long) and generative hyphae always with clamp connections (Malysheva and Spirin 2017).

In regard to previously described Australian species against which *H.australiense* should be compared, the coriaceous, resupinate species of the *Auriculariales* are poorly sampled from Australia. May et al. (2003) listed records from Australia of a number of species of *Eichleriella*, *Exidiopsis* and *Heterochaete* that were originally described from the Northern Hemisphere. Such records remain suspect unless confirmed. Only two new species have been described on the basis of type materials from Australia that may fall within these three genera: *Heterochaetecheesmanii* Wakef. and *Irpexdepauperatus* Massee.

*Heterochaetecheesmanii* was described by Wakefield (1915) from a collection on wood from New South Wales, characterized by the thin, orbicular basidiomes with a shortly reflexed margin, the pale hymenium with sparse, minute spines, the soft fulvous context, with 4-spored, cruciate basidia 15 × 10–12 μm, and curved, cylindrical spores, 14–15 × 5–5.5 μm, and hyphae 1.5–4 μm diameter. Reid (1957) examined the type at K and noted the presence of “conspicuous branched paraphyses”. *Heterochaetecheesmanii* differs from *H.australiense* by the shorter basidiospores. It will be necessary to obtain sequences from *H.cheesmanii* to ascertain its correct generic placement, but it could well be a member of *Heteroradulum*.

*Irpexdepauperatus* was introduced by Massee (1901) with a short description, based on a collection on dead bark by Rodway from Tasmania. Note that due to existence of the previously described *Irpexdepauperatus* Berk. & Broome, the replacement name *Irpextasmanicus* Syd. & P. Syd. was introduced for *I.depauperatus* Massee. According to Massee (1901), *Irpexdepauperatus* Massee was characterized by the tawny hymenium with short, laterally incised spines forming orbicular then confluent patches with a white edge and basidiospores of 6 × 3–4 μm. No comparison against other species was provided in the protologue. Both Bodman (1952) and Reid (1957) placed *I.depauperatus* as a synonym of other species. Without examining the type, Bodman (1952) listed *I.depauperatus* as a possible synonym of *Heterochaetedelicata* (Klotzsch) Bres. However, Reid (1957) considered that *I.depauperatus* was a synonym of *Eichleriellaspinulosa* (Berk. & M.A. Curtis) D.A. Reid (basionym *Radulumspinulosum* Berk. & M.A. Curtis, now accepted as *Heteroradulumspinulosum*). Reid (1957) provided a description of *E.spinulosa* (with *I.depauperatus* listed as synonym) that is evidently based on the cited Australian specimen (*Miller s.n.*, K, Herb. F.P.S.M. No. 4996). Despite the fact that Massee (1901) originally described *I.depauperatus* as having basidiospores of 6 × 3–4 μm, Reid (1957) found that the type at K has basidiospores of 19 × 7 μm, matching the basidiospores from the Australian collection by Miller in 1954, but he did not provide any further details of the characters of the type collection of *I.depauperatus*.

*Irpexdepauperatus* potentially belongs in *Heteroradulum* but due to slight morphological differences between species such as *H.australiense* and *H.spinulosum*, and the potential for further species to occur in the region, DNA sequences would be ideal to assist in interpretation of the old name. However, it is unlikely to be able to readily obtain DNA from the more than 100-year old type of *Irpexdepauperatus*, which is borne out by unsuccessful attempts to amplify ITS and LSU sequences from several Australian collections in MEL filed under *Heterochaete*, collected in the 1950s and 1960s. Collections for which DNA amplification was unsuccessful included MEL 2313650 (which is a duplicate of the K collection *Miller s.n.*, Herb. F.P.S.M. No. 4996). The morphology of *Miller s.n.* as recorded by Reid (1957) matches *H.australiense* in basidiospore size and shape and presence of skeletocystidia. However, the connection between this collection and the type of *Irpexdepauperatus* is not definite, as only basidiospore dimensions of the latter were provided by Reid (1957). It remains possible that *Irpextasmanicus* (= *I.depauperatus*) represents an earlier name for *Heteroradulumaustraliense*. Given the lack of a sequence from the type and the meagre morphological details available, we choose to introduce a new species, well-characterized by the combination of morphology and sequence data. Perhaps with the application of next generation sequencing, it may become possible to recover sequences from older types more routinely as has been done already in some cases, such as by Delgat et al. (2019).

### ﻿ Heteroradulum labyrinthinum

Taxon classificationFungiAuricularialesExidiaceae

(H.S. Yuan & C. Decock) L.W. Zhou comb. nov.

5DE9D5F8-D30A-5F0F-A655-CA671CF551AC

842486

#### Basionym.

*Grammatuslabyrinthinus* H.S. Yuan & Decock, in Yuan, Lu & Decock, MycoKeys 35: 32 (2018)

#### Remarks.

*Heteroradulumlabyrinthinum* was placed in the new genus *Grammatus* as the generic type (Yuan et al. 2018). The main reason for introducing *Grammatus* was its irregularly irpicoid to poroid hymenophores, from a morphological perspective (Yuan et al. 2018). However, the morphological difference of hymenophores is not a reliable taxonomic character at the generic level within the *Auriculariales*. For example, *Protomerulius* Möller was recently shown to accommodate species with various kinds of hymenophore (Spirin et al. 2019a). This phenomenon also occurs in other groups of wood-inhabiting fungi (Wang et al. 2021). Moreover, taking the current phylogenetic evidence into consideration (Figure 1), we propose to treat *Grammatus* as a later synonym of *Heteroradulum*. Therefore, *G.labyrinthinus* is transferred to *Heteroradulum*, and *Heteroradulumsemis*, that was moved to *Grammatus* (Yuan et al. 2018), is reaccepted as a member of *Heteroradulum*.

##### ﻿Species excluded from *Heteroradulum*

### ﻿ Heteroradulum yunnanense

Taxon classificationFungiAuricularialesExidiaceae

C.L. Zhao [as ‘ yunnanensis’], in Guan, Liu, Zhao & Zhao, Phytotaxa 437(2): 57 (2020)

BCEEE69B-3337-5F48-82E0-177A8D419C63

#### Remarks.

*Heteroradulumyunnanense* has a white to gray hymenophore and colorless hyphae (Guan et al. 2020), which do not fit well with the concept of *Heteroradulum* sensu Malysheva and Spirin (2017). According to the current phylogenetic evidence, we propose to exclude *H.yunnanense* from *Heteroradulum*.

## ﻿A key to species of *Heteroradulum*

**Table:** 

1	Hymenophore irpicoid to poroid	***H.labyrinthinum***
–	Hymenophore grandinioid to odontioid	**2**
2	Hyphal system monomitic	**3**
–	Hyphal system dimitic	**4**
3	Basidiospores up to 14.2 μm long	***H.adnatum***
–	Basidiospores up to 20.4 μm long	***H.deglubens***
4	Basidiomes perennial	***H.kmetii***
–	Basidiomes annual	**5**
5	Skeletocystidia present	**6**
–	Skeletocystidia absent	**7**
6	Generative hyphae septa with or without clamp connections	***H.australiense***
–	Generative hyphae septa with clamp connections	***H.spinulosum***
7	Cystidia absent	***H.brasiliense***
–	Cystidia present	**8**
8	Basidiospores more than 15 μm long	***H.lividofuscum***
–	Basidiospores less than 15 μm long	***H.semis***

## ﻿Discussion

In this study, the circumscription of *Heteroradulum* is emended by merging the genus *Grammatus*, adding the newly described species *H.australiense* and excluding the species *H.yunnanense*.

Recently, the concept of *Protomerulius*, another genus of the *Auriculariales*, was redefined to accommodate species bearing smooth, poroid and spiny hymenophores (Spirin et al. 2019a). The merging of *Grammatus* into *Heteroradulum* further indicates that while hymenophoral characters may be used to distinguish species they are not reliable characters at genera rank within the *Auriculariales*. In the case of the highly diverse macromorphological characters of species within the *Auriculariales*, the generic and, especially, familial delimitations should be cautiously explored with the aid of as comprehensive phylogenetic samplings as possible. Ideally, the construction of an order-level phylogenetic framework with wider taxon sampling and multimarker sequencing will help exactly clarify the higher-level relationships.

*Heteroradulumyunnanense* was placed in *Heteroradulum* based on a quite simple phylogeny with limited samples (Guan et al. 2020). Guan et al. (2020) stated that *H.yunnanense* grouped together with *H.adnatum*, but it actually was separated from all sampled species of *Heteroradulum*. The improper selection of outgroup taxa and absence of additional ingroup taxa lead to the inaccurate taxonomic placement of *H.yunnanense*. In the current phylogeny, *H.yunnanense* has a closer relationship with *Exidiopsiscalcea* (Pers.) K. Wells, *E.grisea* (Bres.) Bourdot & Maire (TUFC100049) and an unnamed taxon of *Exidiopsis* (Figure 1). However, the generic type of *Exidiopsis*, *E.effusa*, is separated from the three so-called taxa of *Exidiopsis*. Consequently, it is premature to transfer *H.yunnanense* to another genus at this stage, but it clearly does not belong in *Heteroradulum*. A wider sampling of species related to *H.yunnanense* and disposition of species of *Exidiopsis* not conspecific with the type is needed to reveal its taxonomic position at a generic level.

## Supplementary Material

XML Treatment for Heteroradulum

XML Treatment for Heteroradulum australiense

XML Treatment for Heteroradulum labyrinthinum

XML Treatment for Heteroradulum yunnanense
